# Induction of tumor initiation is dependent on CD44s in c-Met^+^ hepatocellular carcinoma

**DOI:** 10.1186/s12885-015-1166-4

**Published:** 2015-03-21

**Authors:** Hien Dang, Steven N Steinway, Wei Ding, Carl B Rountree

**Affiliations:** 1 Department of Pediatrics and Pharmacology, The Pennsylvania State University, College of Medicine, Penn State Children’s Hospital, Hershey, PA USA; 2 Laboratory of Human Carcinogenesis, Center for Cancer Research, National Cancer Institute, 37 Convent Drive, Bethesda, MD 20892 USA; 3 Bon Secours St. Mary’s Hospital, 5875 Bremo Road, MOB South Suite 303, Richmond, VA 23226 USA

## Abstract

**Background:**

Hepatocellular carcinoma (HCC) patients with active hepatocyte growth factor (HGF)/c-Met signaling have a significantly worse prognosis. c-Met, a high affinity receptor for HGF, plays a critical role in cancer growth, invasion and metastasis. c-Met and CD44 have been utilized as cell surface markers to identify mesenchymal tumor-initiating stem-like cells (TISC) in several cancers including HCC. In this work, we examine the complex relationship between c-Met and CD44s (standard form), and investigate the specific role of CD44s as a tumor initiator and stemness marker in HCC.

**Methods:**

Gene and protein expression assays were utilized to investigate the relationship between CD44s and c-Met in HCC cell lines. Tumor-sphere assays and *in vivo* tumor assays were performed to investigate the role of CD44+ cells as TISCs. Student’s *t*-test or one-way ANOVA with Tukeys post-hoc test was performed to test for differences amongst groups with a p < .05 as significant.

**Results:**

In an immunohistochemical and immunoblot analysis of human HCC samples, we observed that more than 39% of human HCC samples express c-Met and CD44. To study the relationship between c-Met and CD44, we used MHCC97-H cells, which are CD44^+^/c-Met^+^. The knockdown of c-Met in MHCC97-H cells decreased CD44s, reduced TISC characteristics and decreased tumorsphere formation. Furthermore, we demonstrate that the inhibition of PI3K/AKT signaling decreased CD44s expression and subsequently decreased tumorsphere formation. The down-regulation of CD44s leads to a significant loss of a TISC and mesenchymal phenotype. Finally, the down-regulation of CD44s in MHCC97-H cells decreased tumor initiation *in vivo* compared with the scrambled control.

**Conclusions:**

In summary, our data suggest that CD44s is modulated by the c-Met-PI3K-AKT signaling cascade to support a mesenchymal and TISC phenotype in HCC cells. Moreover, c-Met could be a potential therapeutic drug for targeting HCC cells with TISC and mesenchymal phenotypes.

**Electronic supplementary material:**

The online version of this article (doi:10.1186/s12885-015-1166-4) contains supplementary material, which is available to authorized users.

## Background

Hepatocellular carcinoma (HCC) is the third leading cause of cancer related deaths worldwide [1]. Evidence suggests that HCC arises as a direct consequence of dysregulated proliferation of hepatic progenitor cells [2,3]. Such progenitors, called tumor-initiating stem-like cells (TISCs), have been described in many different malignancies, including HCC, and may account for poor survival and chemotherapy resistance within specific tumors [4,5]. Transcriptome analysis of HCC has demonstrated that a progenitor-based (TISC-phenotype) expression profile is associated with a poor prognosis compared with differentiated tumors (hepatocyte-phenotype) [6-8]. TISCs exhibit the capacity for rapid tumorsphere formation, enriched stem cell gene expression profile, and efficient tumor initiation *in vivo*. Moreover, recent evidence suggests that TISCs have mesenchymal features such as low expression of E-cadherin and high expression of Fibronectin and Zeb1 [9]. Furthermore, TISCs share multiple gene networks involved in self-renewal (i.e. increased expression of stem cell markers such as NANOG, POU5F1 and BMI-1), drug efflux or resistance to chemotherapy drugs, survival, and pluripotency with embryonic stem cells [9,10].

c-Met is a receptor tyrosine kinase that, upon activation by its ligand hepatocyte growth factor (HGF), promotes malignant progression and metastasis in multiple cancers, including HCC [11,12]. Interestingly, 40% of HCC cases are c-Met^+^, and c-Met expression is associated with a poor prognosis [11,13,14]. Aberrant c-Met activation can occur through multiple mechanisms, including autocrine or paracrine ligand-dependent stimulation, mutational activation or gene amplification [12]. During development, homozygous deletion of HGF or c-Met is embryonic lethal [15,16]. Although HGF/c-Met signaling does not play a role in liver homeostasis during normal physiologic conditions, many studies have demonstrated the important role of HGF in liver regeneration, hepatocyte survival, and tissue remodeling after acute injury. Following c-Met phosphorylation and activation, multiple signaling pathways are involved as downstream targets, such as the PI3K/AKT and MAPK/ERK1/2 pathways [17,18].

CD44 is a transmembrane cell adhesion glycoprotein that participates in many cellular processes, including the regulation of cellular growth, survival, differentiation, lymphocyte homing, and motility [19,20]. The variety of cellular processes affected by CD44 is likely the result of multiple CD44 isoforms produced by alternative splicing [21-23]. CD44s, the smallest (standard) form of CD44 (CD44s) is approximately 80–95 kD and lacks all CD44 variable exons. In breast cancer, cells undergoing EMT exhibit increased CD44 expression and TISC characteristics [24,25]. Although, CD44 expression has been described within TISC populations, the isoform responsible for the TISC characteristics remain unclear [20]. CD44s is the predominant CD44 variant, which is ubiquitously expressed in epithelial tissues, and has recently been proposed to be essential for epithelial-to-mesenchymal transition (EMT) [26]. Recent studies demonstrate that the RNA binding protein IMP3 stabilizes CD44 mRNA to facilitate cell migration and more importantly, CD44s combined with IMP3 can serve as a biomarker in predicting HCC [27]. Together, these studies suggest the important role of CD44s in HCC progression.

CD44^+^/c-Met^+^ cells have been demonstrated to be tumorigenic with stemness characteristics in pancreatic cancer, which suggests a dual role of c-Met and CD44 as regulators of tumor initiation [28]. More recently, c-Met + inhibitors have been demonstrated to improve overall survival of advanced HCC patients [12]. Thus, understanding how c-Met elicits its oncogenic activities is important in the development of HCC therapies. Using HCC cell lines, we have previously demonstrated that pharmacologic inhibition of c-Met results in the decreased expression of CD44, which indicates a potential link between CD44s and c-Met activation [11]. In the current study, we investigate the co-regulation of c-Met and CD44s. Here, we define a specific functional role of CD44s as a tumor-initiating regulator in HCC. Our results demonstrate that c-Met regulates tumor initiation and mesenchymal stemness features through the activation of PI3K/AKT/CD44s cascade. Our study provides insight on how c-Met + HCC may be resistant to standard chemotherapy, implicating the importance of precision medicine to improve overall survival in HCC patients.

## Methods

### Cell culture

The human HCC cell line MHCC97-H was provided by Dr. Xinwei Wang, from the National Cancer Institute (NCI), under agreement with the Liver Cancer Institute, Zhongshan Hospital, Fudan University, Shanghai, China, and was cultured as previously described [11,29]. The human HCC cell lines Huh7 and Hep3B [acquired from AddexBio (San Diego, CA)] were maintained as previously described [30]. The human SK-Hep1 cells were provided by Dr. Brian Barth, Penn State College of Medicine, and maintained in Dulbecco’s modified Eagles Medium 1X supplemented with 10% defined FBS (Hyclone Laboratories, Logan, UT), 1 mM GlutaMAX-1 (Life Technologies), 100 U/ml penicillin and 100 μg/ml streptomycin. The cells were cultured in a humidified incubator with 5% CO_2_ at 37°C.

### siRNA and shRNA plasmid constructs and generation of stable cell lines

c-Met siRNA was acquired from Thermo Scientific (Dharmacon, Chicago, IL). Stable shRNA: TG320418 HuSH 29mer shRNA constructs against c-Met in the pGFP-V-RS vector was purchase from OriGene (Rockville, MD). The following constructs have been validated using real-time PCR assays and have been used for developing stable c-Met knock-down cell line. The c-Met shRNA targeting sequence of construct 1: 5′-TACTGCTGACATACAGTCGGAGGTTCACT-3′ and construct 2: 5′-ACACTCCTCATTTGGATAGGCTTGTAAGT-3′. The scrambled shRNA construct with the pGFP-V-RS backbone was purchased from OriGene (Cat# TR30013). Short-hairpin construct oligonucleotide inserts of CD44s were generated for the psiRNA-h7SK G1 (clone sites: Bbsl/Bbsl) expression vector. Sequencing was performed to verify the presence of the siRNA. The CD44s shRNA targeting construct was 5′-CAAGTGGACTCAACGGAGA-3′. MHCC97-H cells were transfected with either scrambled shRNA, c-Met shRNA, or CD44s shRNA using Fugene 6 transfection reagents per manufactures recommendation (Promega, Sunnyvale, CA). Twenty-four hours after transfection, puromycin (2 μg/ml) was added to select stable c-Met shRNA clones, and 100 μg/ml of zeocin was added to select stable CD44s clones. Multiple pooled clones of stable MHHCC97-H cells containing scrambled shRNA and CD44s shRNA and single clones containing c-Met shRNA were isolated and expanded. Knock-down of c-Met and CD44s expression was validated using both real-time PCR and western blot assays as previously described [31,32].

### qRT-PCR

RNA isolation and quantitative polymerase chain reaction experiments were performed as previously described [24].

### Western blot analysis

c-Met, phospho-c-Met (1349), phospho-c-Met (1234/1235), AKT, phospho-AKT, ERK1/ERK2, phospho-ERK1/ERK2, CD44, E-cadherin, vimetin, and moesin antibodies were purchased from Cell Signaling Technology (Danvers, MA). β-actin antibody was obtained from Sigma (Allentown, PA). CD44v6 was obtained from eBioscience (San Diego, CA) and fibronectin was obtained from BD Sciences (San Jose, CA). All human HCC samples were obtained through an IRB approved protocol (IRB#27146). Tissue samples were incubated with lysis buffer and incubated on ice for 10 min followed by disruption by the TissueLyser (Qiagen, CA) per manufacturer’s recommendation. 15-30 μg of cell lysates were collected and western blot was performed as previously described [30]. For densitometry analysis, scanned blots were analyzed using Image J (v1.48. NIH, Bethesda, MD) and normalized to Beta-Actin loading control after background subtraction.

### Microarray analysis

Using the MHCC97-H CD44s shRNA, MHCC97-H c-Met shRNA or MHCC97-H scrambled shRNA cells, mRNA was hybridized to an Illumina human gene chip as previously described by the Penn State Functional genomics core [33]. Experiments were performed in triplicates. Housekeeping genes were used as standards to generate expression levels, and data analysis was conducted using 1.4-fold or greater change in expression with P < 0.05 as significant. The full complement of the expression data is available at http://www.ncbi.nlm.nih.gov/geo (accession number GSE38343).

### Spheroid formation assay/cell viability assays

The capability of self-renewal and cell viability assays were assessed as previously described [24]. Briefly, 1X10^5^ MHCC97-H cells were transfected with 25pM of c-Met siRNA or scrambled siRNA (Thermo Scientific, Dharmacon, Chicago, IL) followed by transfection of pBabe CD44s construct or pBabe EV and incubated for an additional 24 hours. The cells were counted with trypan blue exclusion and 5x10^3^ cells were plated onto low adherent 6-well plates for an additional 2 weeks.

### Animal care and xenograft transplantation experiments

Nude Mice (Jackson Laboratory, Bar Harbor, ME) were fed and housed as previously described [11]. All of the procedures were in compliance with our institution’s guidelines for the use of laboratory animals and approved by the Penn State Institutional Animal Care and Use Ethics Committee. The cells were counted with trypan blue exclusion and suspended in a 1:3 dilution of Matrigel (Matrigel: DMEM/F12 supplemented with 10% FBS). Three different cell dilutions were used for bilateral subcutaneous injection: 1X10^4^ cells/100 mL, 1X10^3^ cells/100 mL and 1X10^2^ cells/100 mL. Serial diluted cells were inoculated into 10-week-old female nude mice (Jackson Laboratory, Bar Harbor, ME). Tumor initiation was checked every 3–4 days after injection. Caliper measurements of tumor volume (length × width × height) were conducted at the end of the study. The mice were sacrificed, and tumor tissues were fixed for histology studies or frozen for protein extraction.

### Statistical analysis

Microarray statistical analysis was performed as describe [33]. Student’s *t* test was used comparing two groups. One-way ANOVA was used when comparing multiple groups followed by Tukeys post-hoc test to look for differences amongst groups. All analysis with a p < 0.05 was considered statistically significant.

### Immunohistochemistry

Paraffin embedded slides were labeled with anti-CD44 (Cell Signaling, Danvers, MA) and anti-c-Met antibodies (Cell Signaling) and stained as previously described [11]. Slides were scored positive if CD44 or c-Met staining were >10% positive for each sample. HD and SS scored all IHC samples. Only samples that were considered positive by both HD and SS were used for statistics.

### Flow cytometry (FACS) analysis

FACS experiments were performed using one million cells, incubated with mouse anti-human CD44-PE (BD Biosciences, Falcon Lakes, NJ) or anti-human c-Met/2-APC (eBiosciences, San Diego, CA). Analysis was performed using a FACS Calibur (BD Biosciences, Falcon Lakes, NJ). Post-FACS analysis was performed using the Flow-Jo program (Tree Star, Ashland, OR). Positive and negative gates were determined using immunoglobulin G (IgG)-stained and unstained controls.

## Results

### CD44 expression correlates with c-Met expression in human HCC

**Figure 1:**
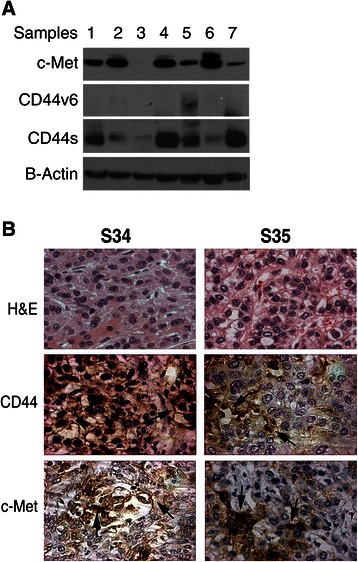
**CD44s correlates with c-Met expression in human HCC samples. (A)** Representative western blot in which 7 out of 33 human clinical HCC samples demonstrating c-Met, CD44v6 and CD44s co-expression. See Additional file 1: Figure S1 for all 33 samples. **(B)** Representative images of CD44 and c-Met immunohistochemistry performed on 68 human HCC tissues using anti-CD44 and c-Met antibodies (400X).

To investigate the correlation between c-Met and CD44, we performed immunohistochemistry staining on 68 HCC tumors (Figure 1B) and immnoblotted 33 HCC tumors (Figure 1A). Immunohistochemical analysis demonstrated that 39% (27/68) of the human HCC samples are c-Met^+^ CD44^+^ (Figure 1B). Immunoblot analysis of an additional 33 HCC samples demonstrated a similar correlation between c-Met and CD44s in 45% (15/33) of the samples (Figure 1A and Additional file 1: Figure S1).

### c-Met^+^CD44s^+^ HCC cells have increased mesenchymal characteristics

**Figure 2:**
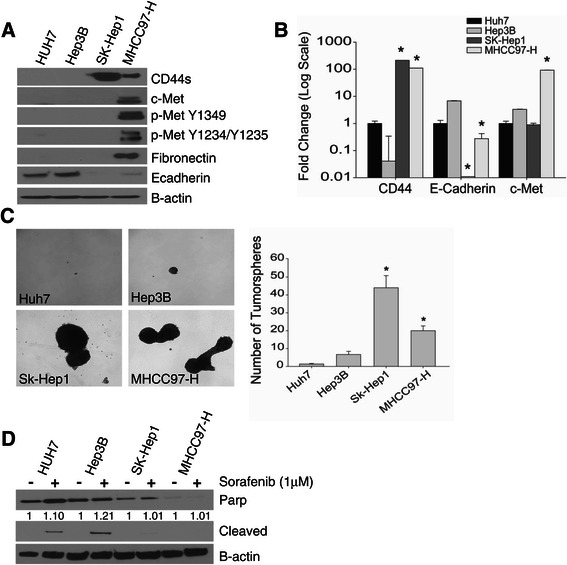
**CD44s**^**+**^**HCC cells have mesenchymal and tumor-initiating stem-like characteristics. (A)** Protein expression of endogenous CD44s, c-Met, and mesenchymal markers. The data are representative of three independent experiments. **(B)** Relative mRNA expression of mRNAs encoding CD44, E-cadherin and c-Met normalized to the endogenous control GAPDH. Data represent mean ± SEM of triplicates, *p < 0.01. **(C)** Tumorsphere assay was performed for two weeks in non-adherent culture plates and the numbers of tumorspheres were counted. The data represent the mean ± SEM of triplicates, *p < 0.01. Phase-contrast images are representative of triplicates (40X magnification). **(D)** HCC cells were treated with sorafenib for 24 h. Endogenous protein expression of apoptotic markers PARP, cleaved PARP, and B-actin were measured via western blotting. Presented densitometry values represent relative expression relative to total PARP after normalization to B-actin.

To study the potential relationship between CD44s and c-Met in HCC, we characterized four human HCC cell lines: Huh7, Hep3B, Sk-Hep1 and MHCC97-H. Flow cytometry analysis demonstrates that both the SK-Hep1 and MHCC97-H cell lines are 99% CD44^+^ compared with the Huh7 and Hep3B cells, whose CD44^+^ cell proportions are less than 1.5% (Additional file 2: Figure S2A). Further characterization of the four cell lines demonstrate that CD44^+^ cell lines can readily form tumorspheres, have a mesenchymal phenotype with decreased E-cadherin, and have resistance to sorafenib and doxorubicin chemotherapy treatment (Figure 2A-D) and Additional file 2: Figures S2B-C). The MHCC97-H cells demonstrated increased expression of both CD44 and c-Met; thus, the MHCC97-H cells provide the best model for the c-Met^+^/CD44^+^ HCC phenotype that has been observed in human HCC samples.

### c-Met regulates TISC characteristics, mesenchymal features, and CD44s expression

**Figure 3:**
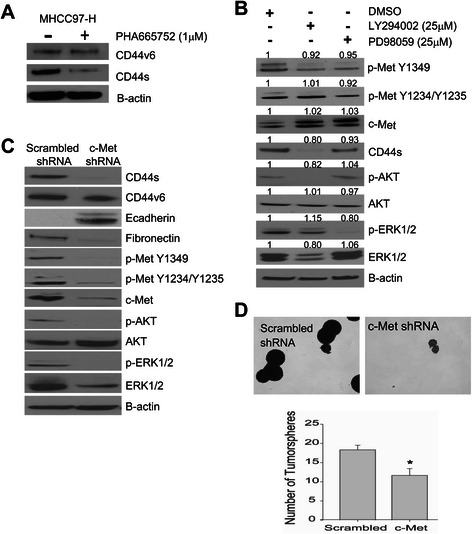
**c-Met regulates CD44s expression through AKT signaling. (A)** MHCC97-H cells were treated with 1 μM of PHA665752 for 24 h. Immunoblot analysis of CD44v6 (~160 kDa), CD44s (~85 kDa), and B-actin. **(B)** MHCC97-H cells were treated with 25 μM DMSO, LY294002 (PI3K inhibitor) or PD98059 (MEK inhibitor) for 24 h, and immunoblot analysis was performed. **(C)** MHCC97-H cells were stably transfected with c-Met shRNA. Lysates were collected after 2 passages and CD44s, fibronectin, and E-cadherin expression was via immunoblotting. **(D)** Tumorsphere formation (40X magnification) assay of stably transfected MHCC97-H cells with c-Met shRNA compared to scrambled control. The data represent the mean ± SEM of triplicates, *p < 0.01. Phase-contrast images are representative of triplicates (40X magnification).

We have previously demonstrated that pharmacologic inhibition of c-Met in MHCC97-H cells results in a reduction of tumor growth and decreased CD44 expression [11]. Moreover, previous studies have demonstrated that CD44v6 interacts with c-Met to enhance downstream MET activation. Therefore, we wanted to test whether CD44v6 was modulated by c-Met inhibition. Interestingly, inhibition of c-Met by PHA66572, a selective inhibitor of c-Met, had a greater effect on CD44s (approximately 85-95kDA) than CD44v6 (approximately 160kDA) (Figure 3A).

To test how c-Met regulates CD44s, we individually targeted PI3K/AKT or MAPK/ERK1/2 pathways using the small molecule inhibitors LY294002 and PD798059, respectively. CD44s expression was significantly decreased after PI3K inhibition (LY29402) compared with vehicle (DMSO) control but only a slight change with MAPK inhibition (PD798059, Figure 3B). This suggests that CD44s expression is regulated by the c-Met-PI3K-AKT signaling.

To further investigate the relationship between c-Met and CD44s, we developed a stable MHCC97-H c-Met shRNA cell line (Figure 3C). In the MHCC97-H c-Met shRNA cells, PI3K/AKT, MAPK/ERK1/2 signaling, and CD44s expression are down-regulated compared to control cells. Furthermore, the down-regulation of c-Met leads to increased E-cadherin expression, decrease Fibronectin expression and decreased tumorsphere formation (Figure 3C and D).

### CD44s regulates TISC and mesenchymal characteristics

**Figure 4:**
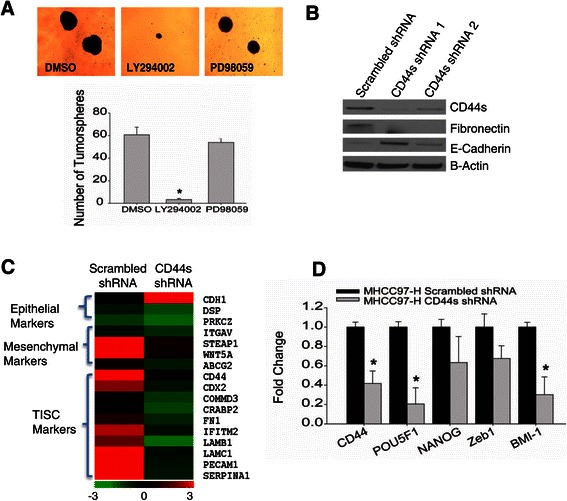
**CD44s regulates mesenchymal and tumor-initiating stem-like characteristics. (A)** Tumorsphere assay of MHCC97-H cells treated with DMSO, LY294002 or PD98059 (25 μM) for 24 h followed by trypan blue exclusion. 1x10^5 cells were plated in triplicates in 6-well low adherent plates for an additional 2 weeks. The data represent the mean ± SEM of triplicates, *p < 0.05. Phase contrast images are at 40X magnification. **(B)** Endogenous protein levels of CD44s and the mesenchymal markers E-cadherin and Fibronectin in two pooled stable CD44s shRNA cell lines. **(C-D)** Heatmap of MHCC97-H CD44s shRNA compared to scrambled control cells. Relative mRNA expression of stem cell genes in CD44s shRNA #1 compared with the scrambled control normalized to GAPDH. The data represent the mean ± SEM of triplicates, *p < 0.001.

We next wanted to test whether the regulation of TISC and mesenchymal features is through CD44s. To do so, MHCC97-H cells were treated with LY294002 or PD798059 for two weeks in low adherent culture conditions. Accordingly, LY294002 was able to significantly inhibit tumorsphere formation compared to vehicle or PD98059 (Figure 4A), suggesting that the inhibition of PI3K activity and subsequent loss of CD44s could inhibit tumorsphere formation.

The down-regulation of CD44s and the significant decrease in tumorsphere formation after PI3K/AKT inhibition suggests that CD44s may be a critical TISC regulator. To test whether CD44s regulates tumor-initiating characteristics, we generated stable MHCC97-H CD44s shRNA cell lines (Figure 4B) and performed microarray analysis. Compared with the MHCC97-H scrambled shRNA cells, the down-regulation of CD44s decreased the expression of mesenchymal, and TISC markers and increased epithelial markers (Figure 4C). Moreover, qRT-PCR analysis confirmed the significant down-regulation of TISC genes (Figure 4D).

### c-Met activation of mesenchymal and TISC characteristics occurs through CD44s

**Figure 5:**
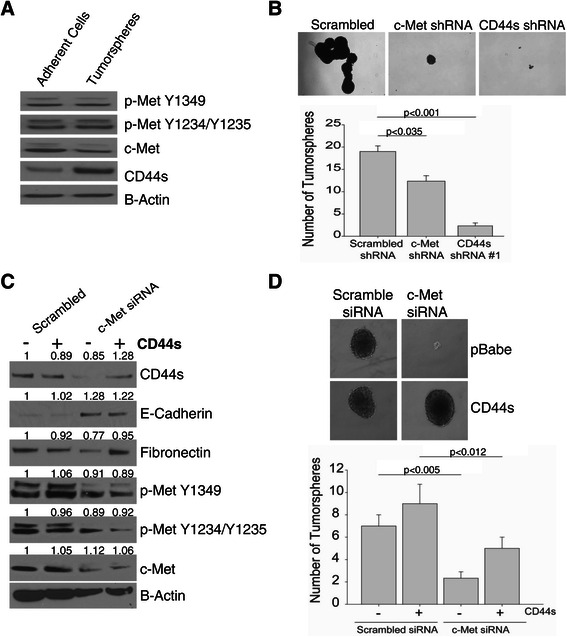
**c-Met induces tumor-initiating characteristics through CD44s. (A)** 5x10^4^ MHCC97-H cells were plated onto 6-well low adherent culture plates in triplicates. Tumorspheres were collected by centrifugation after 2 weeks . For monolayer cultured cells, 5x10^4^ MHCC97-H were plated on 10 cm plate and cultured for 2 weeks. Media was changed every 2–3 days. MHCC97-H cells were plated in low-adherent cell or monolayer cell culture dishes for two weeks and immunoblotting analysis was confirmed on tumorsphere lysates. The data are representative of three independent experiments. **(B)** 5x10^3^ MHCC97-H scrambled, c-Met shRNA and CD44s shRNA cells were grown in 6-well low-adherent culture plates for two weeks and the numbers of tumorspheres were counted (40X magnification). The data are representative of two independent experiments and are shown as the mean ± SEM of triplicate plates. **(C)** CD44s recovers tumorsphere formation. MHCC97-H cells were transfected with c-Met siRNA (25pM) for 24 hrs followed by overexpression of CD44s or pBabe empty vector retrovirus for an additional 48 h (immunoblot) or two weeks (tumorsphere assay). **(D)** Immunoblot data are representative of two independent experiments. The data for the tumorsphere assay data are representative of two independent experiments and are shown as the mean ± SEM of triplicate wells (40X magnification).

Our observations thus far suggest that c-Met regulates CD44s expression through PI3K-AKT signaling to mediate mesenchymal and TISC characteristics. To further investigate the role of CD44s in regulating TISC characteristics, we compared MHCC97-H monolayer cultured cells with tumorspheres. Accordingly, immunoblot analysis shows no difference in c-Met expression and a significant increase in CD44s expression in tumorspheres compared with monolayer cells (Figure 5A). This data support our observations that CD44s is important for tumorsphere formation, one important characteristic of TISCs.

To further confirm that CD44s is required for tumorsphere formation downstream of c-Met, we performed a tumorsphere assay with MHCC97-H scrambled, c-Met and CD44s shRNA stable cell lines. The down-regulation of CD44s significantly decreased tumorsphere formation compared with c-Met or scrambled shRNA (Figure 5B). Next we tested the hypothesis that CD44s can rescue tumorsphere formation after c-Met inhibition. To test our hypothesis, MHCC97-H cells were transfected with c-Met or scrambled siRNA followed by the over-expression of CD44s for 48 hrs. As previously demonstrated, the down-regulation of c-Met decreased CD44s and Fibronection and increased E-cadherin expression (Figure 5C). However, followed by the subsequent over-expression of CD44s, there was an increase in Fibronectin expression. More importantly, CD44s was able to partially rescue tumorsphere formation (Figure 5D). Together, our data suggest that c-Met regulates TISC and mesenchymal features through CD44s via the PI3K-AKT signaling cascade.

### CD44s regulates tumor initiation *in vivo*

**Figure 6:**
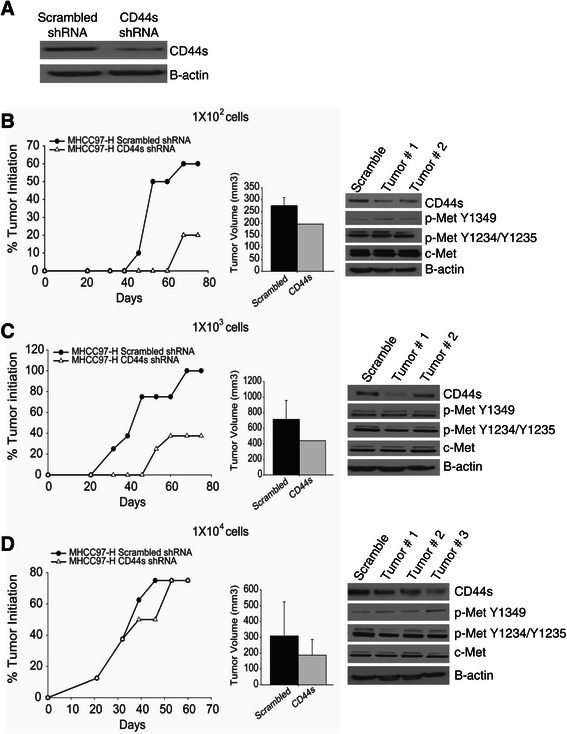
**CD44s regulates tumor initiation*in vivo*. (A)** Immunoblot of MHCC97-H scrambled and CD44s shRNA #1 before cells were injected into athymic nude mice. **(B-D)** Tumor initiation graph of MHCC97-H CD44s shRNA compared with the scrambled shRNA control. Bilateral subcutaneous injections of 1x10^2^, 1x10^3^, or 1x10^4^ cells were inoculated into athymic nude mice, and the number of tumors formed and the percent tumor initiation were calculated (1x10^2^, N = 10; 1x10^3^, N = 8; or 1x10^4^, N = 6). Tumor volume was calculated at the end of the experiment and. Data represent the mean ± SD. Confirmation of down-regulation of CD44s and c-Met signaling was performed by immunoblotting.

To test whether CD44s regulates tumor initiation *in vivo*, we subcutaneously injected athymic nude mice with 1 × 10^2^, 1 × 10^3^, or 1 × 10^4^ MHCC97-H CD44s or scrambled shRNA cells (Figure 6A). Tumor incidence was observed and tumor volume measured at the end of the experiment. Accordingly, the down-regulation of CD44s results in the inhibition of tumor initiation and growth in lower cell dilutions compared to scrambled shRNA controls (Figure 6B-D), an important TISC characteristic.

## Discussion

Hepatocellular carcinoma (HCC), the fifth most common cancer in men and seventh in women, is on the rise in the United States [34]. Due to the diverse etiologies of HCC, including hepatitis B virus (HBV) and hepatitis C virus (HCV) infection, alcoholic diseases and obesity, and its direct impact on the heterogeneity of the tumor, there are limited treatment options with poor survival [35]. Sorafenib is the only FDA approved therapy for advanced HCC, however the benefits are modest [36]. In a randomized clinical trials phase II study, tivantinib, a c-Met inhibitor, has demonstrated to be a promising antitumor agent in c-Met high patients with a median overall survival of more than seven months [37,38]. Notably, we have previously demonstrated that the inhibition of c-Met in c-Met + HCC significantly reduces tumor burden [11]. Together, these studies support the idea that targeted therapy is important for improving the overall survival of HCC patients.

HCC patients with an active c-Met signaling or TISC transcriptome profile have a poor prognosis. In solid tumors, c-Met^+^ and CD44^+^ cells demonstrate increased TISC gene expression profile, increased tumor-sphere formation, and efficient tumor initiation in limited dilution studies [5,28,39-42]. In this study, we demonstrate the underlying mechanism of how c-Met elicits its tumorigenic properties through the activation of CD44s to induce a mesenchymal and TISC phenotype. Although the importance of CD44 in tumor progression and TISC populations has been demonstrated, most reports that define TISC populations with CD44 utilize antibodies that recognizes all CD44 isoforms [20]. However, which CD44 variants are responsible for the TISC phenotypes has yet to be elucidated. In this study, we demonstrate the underlying mechanism of how c-Met elicits its tumorigenic properties through the activation of CD44s in order to induce a mesenchymal and TISC phenotype. Our findings establish for the first time the functional relationship between the CD44 standard variant (CD44s) and c-Met in regulating a TISC phenotype. We confirm that CD44s and c-Met are co-expressed in human HCC by using our own data set [8,43]. We discovered a novel regulatory relationship between CD44s and c-Met that controls mesenchymal and TISC phenotype through the PI3K-AKT signaling pathway.

The relationship between c-Met and CD44v6 is well established [44-46]. Specifically, c-Met regulates CD44 alternative splicing to promote CD44v6 production through RAS signaling [47]. In turn, CD44v6 interacts with c-Met by presenting HGF and subsequently sustains RAS signaling to promote cell proliferation [44,45,47]. This positive feedback loop occurs in an HGF-dependent manner. In the MHCC97-H cells both CD44v6 and CD44s isoforms are expressed. In our work, the down-regulation of c-Met leads to a slight change in CD44v6 expression, suggesting that c-Met may also regulate CD44v6. The question arose as to why cancer cells would express both CD44s and CD44v6 isoforms. This different role of CD44 on c-Met is explained by the difference in CD44 isoforms involved [20]. While CD44v6 amplifies c-Met signaling and cell proliferative through RAS signaling as described by others, our data suggest that c-Met regulates CD44s to promote a mesenchymal and TISC phenotype via the PI3K cascade. While CD44v6 does not play a role in the regulation of a TISC phenotype, it has been demonstrated that CD44v6 is important for cell migration and metastasis by promoting c-Met signaling through ERM (ezrin, radaxin, and moesin) proteins [21,45,48]. By expressing both CD44 isoforms in c-Met + tumors, cancer cells are more likely to be resistant to standard treatment, metastasize, and colonize at distant organ sites. Thus, our current study supports the idea that combination therapy with c-Met inhibitor and CD44 monoclonal antibody may be more effective in anti-tumor activity than c-met inhibition alone. Moreover, the CD44 monoclonal antibody has been demonstrated to be effective in chronic lymphocytic leukemia [49]. Although the role of CD44v6 in cell migration has been well studied in other solid tumors, its functional role in HCC will need to be further investigated.

In this work, we demonstrate the importance of the c-Met/AKT/CD44s cascade in promoting a TISC phenotype. The down regulation of CD44s significantly decreased tumorsphere formation compared with c-Met shRNA cells. However, CD44s was not able to fully rescue tumorsphere formation after c-Met inhibition, suggesting that c-Met may regulate tumorsphere formation independent of CD44s. The c-Met/HGF signaling cascade is important for morphogenesis during embryonic development and organ regeneration by inducing EMT and can be high-jacked by cancer cells to promote metastasis [12,50]. Furthermore, c-Met has been implicated in regulating the stem/progenitor phenotype by transcriptional regulation of stemness factors including NANOG, POU5F1, and Sox2 [42]. Therefore, it is likely that c-Met, through other mechanisms independent of CD44s, can regulate the TISC and mesenchymal phenotype.

Prior studies have demonstrated that the PI3K/AKT signaling cascades promote a mesenchymal phenotype. Studies have suggested that constitutive PI3K/AKT signaling is required for EMT in squamous cell carcinoma, whereas PI3K/AKT signaling is required for TGFβ induced EMT in breast cancer cells [51,52]. Furthermore, TGFβ-induced EMT generates CD44^+^/CD24^−^ TISCs [25]. Here, we provide evidence consistent with previous findings that the PI3K/AKT signaling is a central pathway for a mesenchymal phenotype through the c-Met/PI3K/AKT/CD44s cascade.

## Conclusions

In this study, we demonstrate a positive correlation between CD44s and c-Met in clinical HCC samples and show, for the first time, a functional relationship between CD44s and c-Met within HCC. We present evidence that c-Met regulates CD44s to drive a mesenchymal and TISC phenotype and that the down regulation of CD44s decreases tumor initiation both *in vitro* and *in vivo*. Our data provide insight into how c-Met induces hepatocarcinogenesis and further support the idea that c-Met represents a potential target for the treatment of c-Met + HCC.

## Additional files

Additional file 1: Figure S1. CD44s and c-Met co-expression in Human HCC samples. Immunblot of HCC samples S8-S33 of CD44s, CD44v6, c-Met, phospho-c-Met Y1234/Y1235 and phospho-c-Met Y1349.
Additional file 2: Figure S2. Characterization of human HCC cells. (A) Flow activated cytometry of human HCC cells for CD44 and c-Met. Data represent triplicates and experiments were performed two independent times. (B) Cell viability assay of HCC cells after 24 hours of doxorubicin treatment at indicated doses. Data represent two independent experiments and are shown as mean ± SEM of 8 replicates, **p < 0.05*. (C) Immunoblot analysis for apoptosis after 24 hours of doxorubicin treatment at 2.5 ng/ml. Presented densitometry values represent relative expression relative to total PARP after normalization to B-actin.

## Abbreviations

TISCs: Tumor-initiating stem-like cells
DMEM: Dulbecco’s modified Eagle medium
EMT: Epithelial-to-mesenchymal transition
FBS: Fetal bovine serum
GFP: Green fluorescence protein
IF: Immunofluorescence
IHC: Immunohistochemistry
HCC: Hepatocellular carcinoma
MET: Mesenchymal-to-epithelial transition
PI3K: Phosphoinositide 3-kinase
PTEN: Phosphatase and tensin homolog deleted on chromosome 10
