# Supplementary figures and images for: Induction of tumor initiation is dependent on CD44s in c-Met+ hepatocellular carcinoma
Source: BMC Cancer. 2015 Mar 21;15:161. doi: 10.1186/s12885-015-1166-4 (PMC4380258; doi:10.1186/s12885-015-1166-4)

Dang Supplementary Figure S1

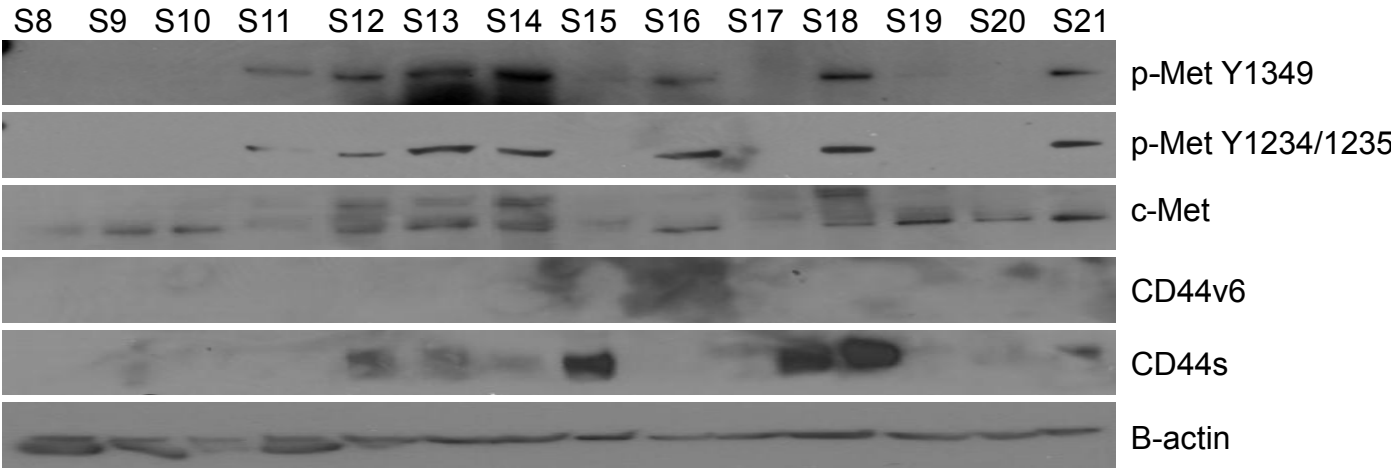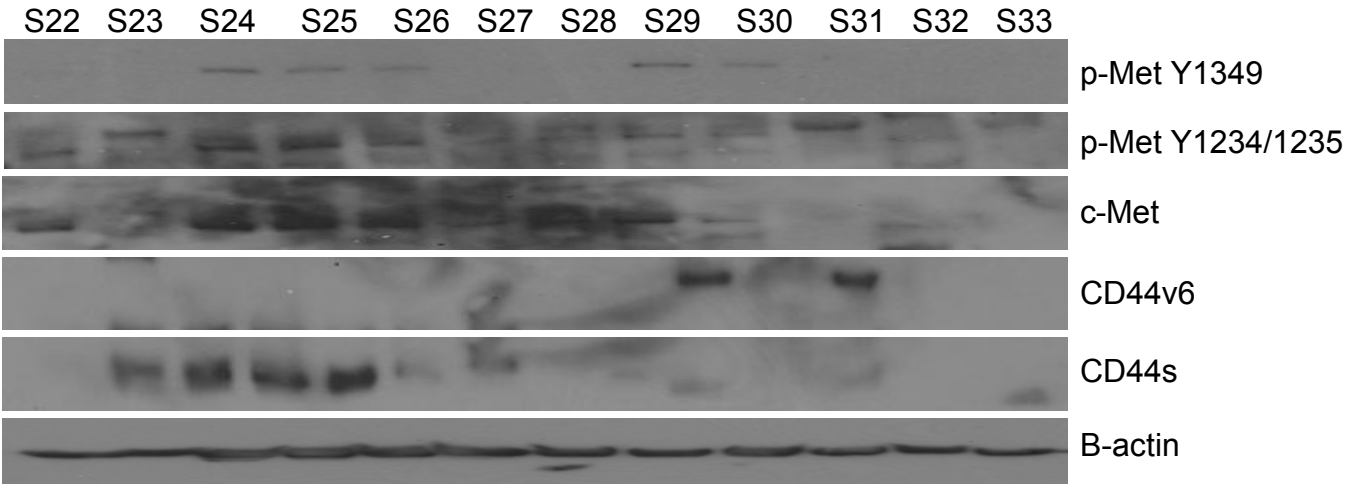

Supplement: Additional file 1: Figure S1. — CD44s and c-Met co-expression in Human HCC samples. Immunblot of HCC samples S8-S33 of CD44s, CD44v6, c-Met, phospho-c-Met Y1234/Y1235 and phospho-c-Met Y1349. [file 12885_2015_1166_MOESM1_ESM.pdf]

A

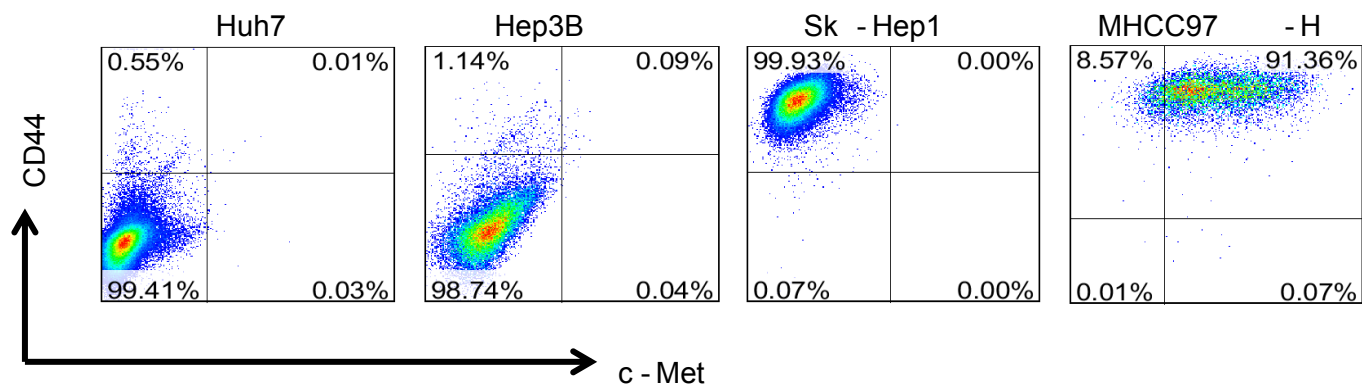

B

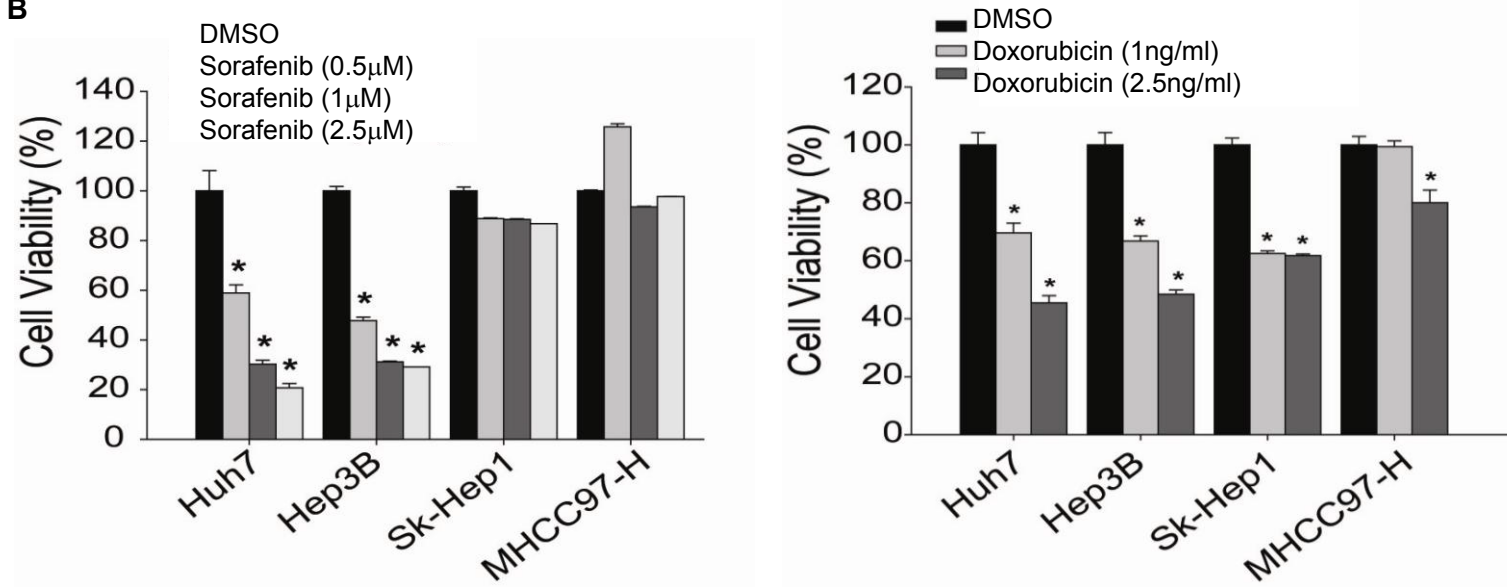

C

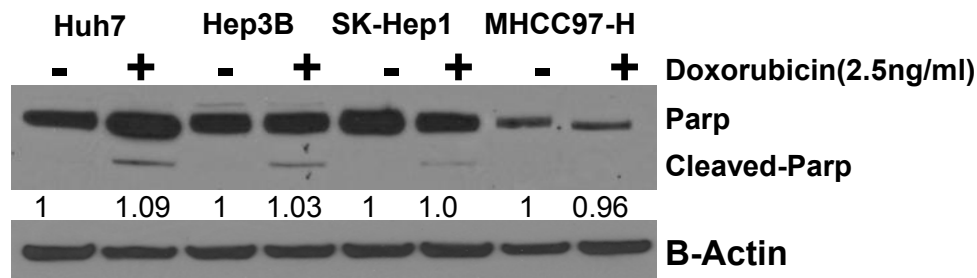

Supplement: Additional file 2: Figure S2. — Characterization of human HCC cells. (A) Flow activated cytometry of human HCC cells for CD44 and c-Met. Data represent triplicates and experiments were performed two independent times. (B) Cell viability assay of HCC cells after 24 hours of doxorubicin treatment at indicated doses. Data represent two independent experiments and are shown as mean ± SEM of 8 replicates, *p < 0.05. (C) Immunoblot analysis for apoptosis after 24 hours of doxorubicin treatment at 2.5 ng/ml. Presented densitometry values represent relative expression relative to total PARP after normalization to B-actin. [file 12885_2015_1166_MOESM2_ESM.pdf]
